# Complete genome sequence of Wildflower, an O cluster mycobacteriophage

**DOI:** 10.1128/mra.00908-24

**Published:** 2024-10-21

**Authors:** Nday Lucie Sungu, Edith Erika Machowski, Christopher Shawn Ealand, Bavesh Davandra Kana

**Affiliations:** 1 DSI/NRF Centre of Excellence for Biomedical TB Research, Faculty of Health Sciences, University of the Witwatersrand, National Health Laboratory Service, Johannesburg, South Africa; Queens College Department of Biology, Queens, New York, USA

**Keywords:** mycobacteriophage, *Mycobacterium smegmatis*

## Abstract

Wildflower is a cluster O mycobacteriophage with a siphoviral morphotype that displays lytic activity in *Mycobacterium smegmatis*. It was isolated from soil in Johannesburg, South Africa. The double-stranded genome consists of 69,364 base pairs with a GC content of 65.5% encoding 121 predicted open reading frames.

## ANNOUNCEMENT

**Fig 1:**
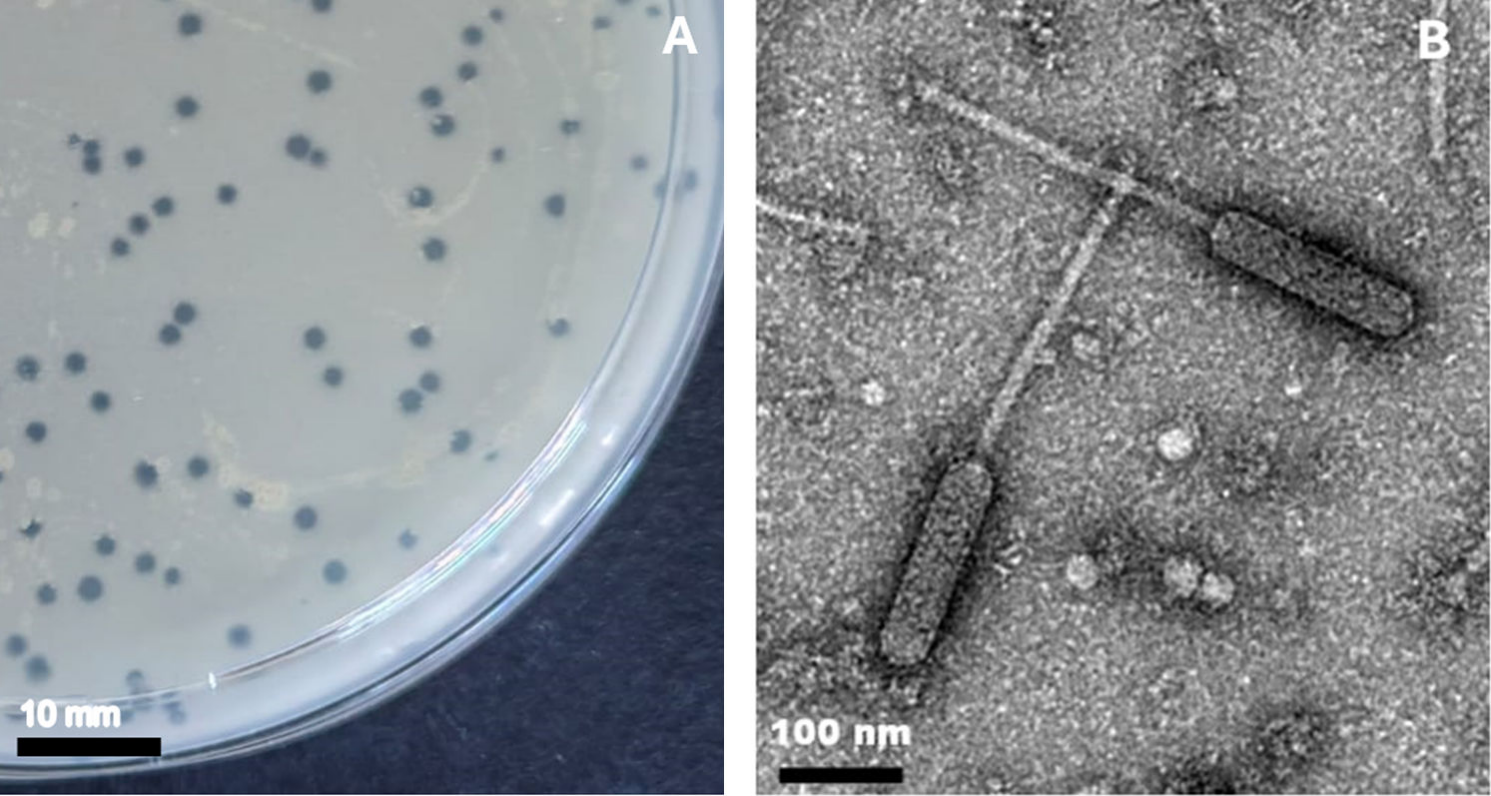
Morphological characterization of mycobacteriophage Wildflower. (**A**) *Mycobacterium smegmatis* mc²155 infected by Wildflower lysate and grown on solid media (7H10) in a petri dish (90 mm). Individual plaques are clear with no visible halo and range from 1 to 2.5 mm in diameter (scale bar 10 mm). (**B**) Transmission electron micrograph of virion morphology (stained with 1% uranyl acetate). Wildflower is comprised of a ~176 nm long × ~47 nm wide head and a ~264 nm noncontractile tail (scale bar = 100 nm).

The persistence of tuberculosis (TB) infections worldwide can be partly attributed to the rise in drug-resistant (DR) bacterial strains (1). Effectively containing DR infections is challenging, as resistance is emerging faster than the development of novel anti-TB drugs (2, 3). Lytic mycobacteriophages potentially widen the repertoire of available anti-bacterial agents and show promise in augmenting established treatment options (4–6). Mycobacteriophage Wildflower was isolated from moist potting soil collected in Johannesburg, South Africa (27 March 2023; GPS coordinates: −26.194718°, 28.032327°). The soil sample was vortexed vigorously in 5 mL of mycobacteriophage buffer (MP) and allowed to settle. The liquid was then filtered-sterilized (0.22 µm) and used to infect *Mycobacterium smegmatis* mc^2^155 (7). Briefly, a 50-µL aliquot of the filtrate was used to infect 450 µL of stationary-phase mc^2^155, previously cultured in 7H9 medium and then washed twice in MP, and finally poured as an overlay on solid media (7H10). Plates were incubated at 37°C for 48–72 hours to obtain plaques (Fig. 1A). One was picked and amplified as described above. High-titer mycobacteriophage lysate was obtained by flooding “lacey plates” with MP buffer followed by harvesting and filter sterilization as previously described (7). High-titer lysate was subsequently used for transmission electron microscopy (TEM) and genomic DNA extraction (Wizard Genomic DNA Purification Kit, Promega). TEM analysis revealed a siphoviral morphotype for Wildflower, with a prolate capsid (~176 nm in length and ~47 nm in width) and ~264 nm long contractile tail (Fig. 1B).

The NEBNext Ultra II FS kit (New England Biolabs) was used for library preparation followed by DNA indexing and sequencing on the Illumina NextSeq500 platform, using a NextSeq (300 cycle) kit as detailed previously (8). A total of 444,230 reads (2 × 150-bp paired end) were generated followed by trimming (Illumina Experiment Manager v1.9 using default settings) before genome assembly. A single mycobacteriophage contig was assembled and assessed for quality, completeness, accuracy, and genomic termini using Newbler (V2.9) and Consed (V29.0) with default parameters. The approximate coverage level was 1,884-fold. Whole-genome nucleotide BLASTn alignments were performed at https://blast.ncbi.nlm.nih.gov/ and https://phagesdb.org/blast/. DNA Master (v5.23.6; http://phagesdb.org/DNAMaster/) was used for genome annotations, with refinements made using supplementary bioinformatics tools, including GeneMark (v2.5p) (9), Glimmer (v3.07) (10), Phamerator (https://phamerator.org/) (11), and HHpred (https://toolkit.tuebingen.mpg.de/tools/hhpred) (12). Wildflower contains a circularly permuted genome of 69,364 bp with a 3′ sticky overhang of 4 bp (GTCT) and a GC content of 65.5%. It shares >98% identity with other cluster O mycobacteriophages, including Dylan (GenBank accession number KF024730), Zakhe101 (KT281796), and Smooch (MN428052). There are 121 predicted open reading frames (ORFs), 80 (61%) of which were annotated as hypothetical proteins. No tRNAs or transfer-messenger RNAs were identified. ORFs with homology to other known genes encode structural elements (e.g., capsid proteins, head-to-tail adaptors, major and minor tail proteins, terminase, and the tape measure protein), elements that facilitate host infection and lysis (e.g., Lysin A and Lysin B and Holin), and DNA-modifying elements (e.g., DNA primase/polymerase and WhiB family transcription factor).

## Data Availability

The Wildflower genome sequence is available at GenBank under accession number PP763603 and the raw sequence reads under BioProject accession number PRJNA1102862.
